# A Novel Design of Tri-Layer Membrane with Controlled Delivery of Paclitaxel and Anti-Biofilm Effect for Biliary Stent Applications

**DOI:** 10.3390/nano11020486

**Published:** 2021-02-14

**Authors:** Abdelrahman I. Rezk, Jeesoo Park, Joon Yeon Moon, Sunny Lee, Chan Hee Park, Cheol Sang Kim

**Affiliations:** 1 Department of Bionanosystem Engineering, Graduate School, Jeonbuk National University, Jeonju 561-756, Korea; rezk@jbnu.ac.kr (A.I.R.); jeesoopark0224@gmail.com (J.P.); mjy34256@naver.com (J.Y.M.); sunny1641@jbnu.ac.kr (S.L.); 2 Department of Bionanotechnology and Bioconvergence Engineering, Graduate School, Jeonbuk National University, Jeonju 561-756, Korea; 3 Division of Mechanical Design Engineering, Jeonbuk National University, Jeonju 561-756, Korea; 4 Eco-Friendly Machine Parts Design Research Center, Jeonbuk National University, Jeonju 54896, Korea

**Keywords:** drug eluting stent, drug delivery, composite nanofiber, bile duct

## Abstract

Here, we developed a novel biliary stent coating material that is composed of tri-layer membrane with dual function of sustained release of paclitaxel (PTX) anticancer drug and antibacterial effect. The advantages of using electrospinning technique were considered for the even distribution of PTX and controlled release profile from the nanofiber mat. Furthermore, film cast method was utilized to fabricate AgNPs-immobilized PU film to direct the release towards the tumor site and suppress the biofilm formation. The in vitro antibacterial test conducted against Gram-positive (*Staphylococcus aureus*) and Gram-negative (*Escherichia coli*) bacteria species showed excellent antibacterial effect. The in vitro drug release study confirmed the sustained release of PTX from the tri-layer membrane and the release profile fitted first order with correlation coefficient of R^2^ = 0.98. Furthermore, the release mechanism was studied using Korsmeyer–Peppas model, revealing that the release mechanism follows Fickian diffusion. Based on the results, this novel tri-layer membrane shows curative potential in clinical development.

## 1. Introduction

Cholangiocarcinoma (CCA), a type of malignancy that develops along the regions from intrahepatic biliary tree to common bile duct, is characterized as the second most common primary hepatic cancer with increasing worldwide incidence [1,2]. Late stage CCA patients commonly exhibit the symptoms of jaundice, cholangitis, and weight loss, but the pathological features vary considerably among those given the same diagnosis [3]. Therefore, treatment strategies vary greatly depending on the stages and metastatic behaviors, ranging from surgical resection to chemotherapy, transarterial embolization, and stenting [4]. Among these treatment options, biliary stents are widely studied and utilized to palliate patients with unresectable malignant tumors. The insertion of nitinol-based bare-metal stents (BMS) across the obstructed site enhances the patients’ bile flow as well as their quality of life [5,6]. Despite the improved patency rate, however, BMS poses unwanted complications that are primarily associated with re-occlusion in the stent strut. In fact, nearly 90% of the stent failures are caused by tumor or epithelial ingrowth and biliary clogging [7]. Compared to BMS, drug-eluting stents (DES) have shown positive outcomes in the treatment of CCA by reducing the risk of restenosis. Most DES targeted for biliary application incorporate chemotherapeutic agents, such as paclitaxel (PTX), bortezomib (BTZ), gemcitabine, and sorafenib, to reduce the proliferation of tumor cells [7,8,9]. Many studies have examined the efficacy of PTX-loaded DES for bile ducts, and with drug concentration of 10% (*w/v*) or lower showed local antitumor activity with minimal adverse effects in the porcine and canine models [10,11]. However, considering that a higher concentration of PTX and direct contact of the drug-loaded membrane in stent struts with the biological tissue may result in inflammatory reactions and other serious complications, proper optimization would be required [12,13,14].

Although loading an antitumor agent can theoretically prolong stent patency, this DES design merely pertains to recurrent obstructions caused by the tumor ingrowth. About 60% of early stent occlusion is caused by biliary clogging and sludge, and continuous effort has been made to address such type of stenting failure with respect to minimizing microbial adhesion and biofilm formation [15,16]. Previous studies suggest that both systemic and local administration of antibiotics and choleretics have little effect in preventing biofilm formation as well as improving the patency [17,18,19]. Many alternative approaches have been proposed to facilitate drainage, ranging from surface modification to immobilization of biomolecules or biocompatible materials [20]. Furthermore, recent development in nanotechnology, such as the application of quorum sensing nano-inhibitors, antimicrobial nanomaterials, and nano-peptides, has offered promising results in managing the microbial colonization in the stent lumen [21,22]. Among the nanomaterials, silver nanoparticles (AgNPs) are widely utilized due to their broad-spectrum antibacterial property, ease of synthesis, and low cost [23,24,25]. Antibacterial effect is initiated as silver ions are released and interact with bacterial walls, creating insufficient environment to form microbial colonies in the bile duct [26,27]. Therefore, we hypothesized that, by incorporating AgNPs into the polymeric membrane of the stent, the matrix would exhibit sufficient antibacterial activity and hinder undesirable biofilm formation in the bile duct. Given that silver contains inherent cytotoxicity at high dose, it is also crucial to assess the biocompatibility in response to the amount of silver nanoparticles loaded onto the membrane.

To our knowledge, no previous research has investigated the combination of drug-eluting and anti-sludge effect for the biliary stent application. In this work, we demonstrate a tri-layer construct and its properties as a biliary stent coating material. Hypothetically, the tri-layer matrix to incorporate into the stent lumen would serve to: (1) control the bacterial adhesion and biofilm generation; (2) inhibit the proliferation of malignant tumor cells by local drug elution while minimizing the side effects; and (3) display sustained drug release profile of PTX while reducing initial burst effect. Therefore, each layer carries a distinctive function that is essential to improving the stent patency as a whole. The inner layer, which is in direct contact with luminal fluid, consists of AgNPs-immobilized PU film with the bacterial biofilm-suppressing effect. Our center membrane is composed of electrospun PU nanofibers loaded with different concentrations of PTX. To control the initial burst release of PTX, we electrospun biodegradable polycaprolactone (PCL) nanofibers on top of the drug-loaded PU membrane. This outermost PCL layer effectively manages the burst release of PTX at the initial stage of stent insertion. Moreover, we hypothesized that the entrapped PTX would have increased exposure as PCL nanofibers degrade over time, thus provoking sustained drug release profile. The series of experiments discussed here were performed to confirm our hypothesis, and comprehensive results in physicochemical characteristics, drug release profile, and antibacterial and cytotoxicity tests demonstrate practical application as a biliary stent coating membrane.

## 2. Experimental

### 2.1. Materials

Poly(ε-caprolactone) (PCL, MW = 70,000–90,000, Sigma-Aldrich, St. Louis, MO, USA), polyurethane (PU, Estane^®^ Skythane X595A-11 TPU, Lubrizol, Cleveland, OH, USA), *N*,*N* dimethylformamide (DMF, 99.5% Samchun, Seoul, Korea), tetrahydrofuran (THF, 99.8% Samchun, Seoul, Korea), silver nitrate (AgNO_3_, Kojima Chemicals CO. LTD., Saitama 350-1335, Japan), and the model drug Paclitaxel (PTX, Samyang Genex Corporation, Gyeonggi-do, Korea) were used.

### 2.2. Film Casting Method

First, 12 wt.% of PU was dissolved in DMF/THF (1:1) and stirred at room temperature overnight, followed by spreading the polymer solution onto a glass plate using knife coating device (KIPAE, KP-3000V, (423-050) Suwon, Korea). The films were dried in vacuum at room temperature for 24 h. For silver-loaded membranes, AgNO_3_ was dissolved in DMF to allow for in situ synthesis of silver nanoparticles via chemical reduction [28]. Then, the solution was added into the second solution of PU and THF. The concentration of silver was adjusted to 1, 3 and 5 wt.% of AgNO_3_ with respect to the polymer weight.

### 2.3. Electrospinning Method

The middle layer, which was electrospun on top of silver-loaded films, was composed of 12 wt.% of PU dissolved in DMF/THF (1:1) and was stirred for 24 h at room temperature. For the outer layer, 12 wt.% of PCL solution was prepared by dissolving weighed amount of PCL pellets in organic solvents (DMF/THF, 1:1) and stirred at room temperature overnight. For this layer, PTX (3 wt.% of the polymer weight) was added to the solution and sonicated for 10 min just before the electrospinning process. Similar to our previous work, electrospinning was conducted under 17 kV of high voltage with the flow rate of 1 mL/h using 21-gauge needle [29].

### 2.4. Characterizations

The morphology of the electrospun nanofiber mats and the films were analyzed by using field-emission scanning electron microscopy (FE-SEM, Carl Zeiss, Supra 40VP, 73447 Oberkochen, Germany). The composition of the surface was investigated using energy dispersive spectrometer (EDS). Phase analysis of the coated film were measured using X-ray diffractometer (XRD, Rigaku, Japan). The chemical interaction between the polymer chains of the different samples were characterized by Fourier transform infrared spectroscopy (FT-IR, Perkin Elmer, Spectrum GX, Waltham, MA 02451, USA) in the range of 4000–400 cm^−1^.

### 2.5. In Vitro Drug Release

The drug release from the nanofiber mat was assessed by placing a known weight of nanofiber membrane into conical tubes containing PBS (10 mL, pH 7.4) and transferring the tubes into a shaking incubator previously set at 37 °C and 100 rpm (SI-300R, Lab companion, (415-850) Kimpo, Gyeonggi-do, Korea) [30]. Then, at different determined time intervals, 3 mL of PBS release media were taken for sampling and put back after the measurement. Using a UV spectrophotometer, the amount of drug release was measured at a wavelength of 232 nm of maximum absorbance of PTX in PBS. Moreover, a syringe device was used to measure the amount of drug diffused from one side, confirming the positive effect of the PCL layer on the controlled drug release (Figure S2). The amount of PTX was determined using a calibration curve constructed from the known PTX concentration. Furthermore, the calibration curve satisfies the Lambert–Beer law:

y = ac + b(1)

To analyze the in vitro release data and evaluate the release kinetics, zero- and first-order kinetic models were used. The zero-order kinetic model defines the drug dissolution from transdermal systems with low soluble drugs, osmotic and coated forms.

(2) $$Q_{t}=Q_{0}+K_{0}t$$

where *Q_t_* is the amount of drug released in time *t*, *Q*_0_ is the initial amount of drug (usually, *Q*_0_ = 0), and *K*_0_ is the zero order release constant (concentration/time). On the other hand, the first-order kinetic model explains the drug release profile of water-soluble drugs in porous matrices, where the drug release is proportional to the concentration of drug remaining in the matrix:

(3) $$\log Q_{t}=\log Q_{0}+\frac{k_{1}t}{2.303}$$

where *Q_t_*, is the amount of drug released in time *t*, *Q*_0_ is the amount of initial drug, and *K*_1_ is the first-order rate constant.

The Korsemeyer–Peppas model describes drug release from a polymeric system:

(4) $$M_{t}/M_{n}=kt^{n}$$

where $M_{t}/M_{n}$ is the fraction of drug release at time *t*, *K* is a rate constant that is dependent on the structural and geometric characteristic of the drug polymer system, and n is the release exponent. The *n* value is used to define different release mechanisms. When the Korsemeyer–Peppas model is applied to thin films, the release exponent *n* ≤ 0.5 corresponds to Fickian diffusion release, while the value of n in the range 0.5 < *n* < 1 is related to anomalous diffusion or non-Fickian release. The results show that drug release follows both erosion mechanisms and diffusion, and *n* = 1 corresponds to Case II where the drug release is independent of time.

### 2.6. Antibacterial Test

The antibacterial activity of Gram-positive *Staphylococcus aureus (S. aureus)* and Gram-negative *Escherichia coli (E. coli)* were investigated against pure PU film and PU films containing 1%, 3%, and 5% of AgNPs (which we refer as PU-Ag 1, PU-Ag 3, and PU-Ag 5, respectively) by inhibition zone testing. Agar plate was prepared with the mixed solution of 1 g tryptone, 0.5 g yeast, 1 g NaCl, 1.5 g agar, and 100 mL DI water sterilized and poured into petri dish (10 mL/each) and allowed to solidify) [31]. Then, bacterial solution diluted to 10^6^ CFU/mL was added and incubated for 24 h. The samples were then placed on the bacteria-containing agar plates. Finally, the plates were incubated at 37 °C for 24 h, and the diameters of the inhibition zone were measured by Image J (NIH, USA) software.

### 2.7. In Vitro Cytotoxicity Test

Prior to seeding CT26 colorectal carcinoma cells (supplied by Korean Cell Line Bank, Seoul, Korea), the scaffolds (PU and PU-PTX-PCL) were sterilized using UV irradiation and 70% ethanol, washed with phosphate buffer saline (PBS 1×, pH = 7.4) three times, and incubated overnight at 37 °C in RPMI, supplied with 10% fetal bovine serum (FBS) and 1% penicillin–streptomycin. The cells were seeded on the substrate at a density of 2 × 10^4^ cells/well and were incubated at 37 °C in the 5% CO_2_ condition. The culture medium was replaced every 24 h. The anticancer drug activity was monitored on Days 1, 3, and 5 using Dojindo’s cell counting kit-8 (CCK-8) assay, where CCK-8 solution was added to each well. After 3 h of incubation at 37 °C, 100 μL of cell suspension were taken out from each well to measure the absorbance at 450 nm using Sunrise microplate reader (Tecan, 5082 Grödig, Austria) [32].

## 3. Results and Discussion

### 3.1. Morphological Characterization and Physicochemical Properties

**Figure 1:**
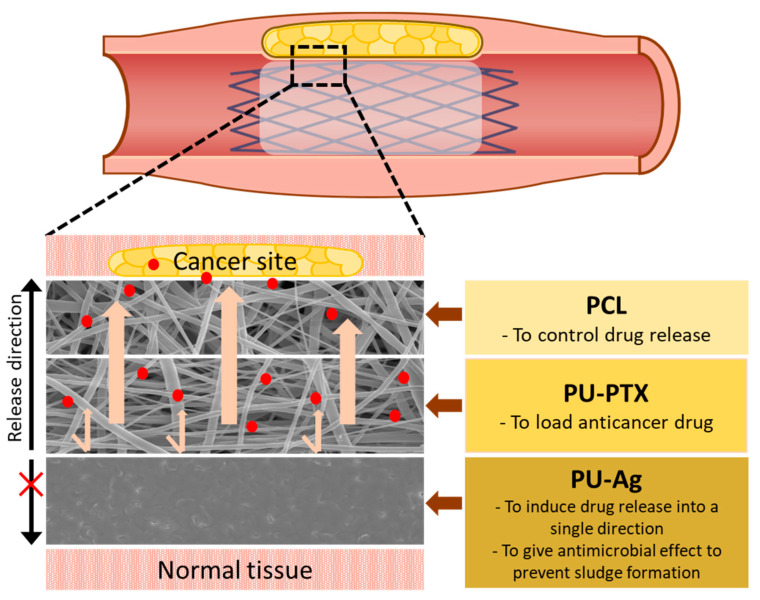
Schematic image of the tri-layer membrane construct for biliary stent application. Each layer provides a different function to maximize stent patency.

Figure 1 shows the schematic diagram describing the tri-layered stent coating design. The innermost layer of PU film loaded with Ag ions is functionally designed to inhibit the biofilm formation on the inner surface of the membrane and to act as a physical barrier to one side to directly guide the drug release toward tumor site. The drug-loaded middle layer consists of PU nanofibers loaded with 3% of PTX while the outer layer of PCL nanofibers functions as a physical barrier as well to sustain the release profile of PTX and improve mechanical properties.

**Figure 2:**
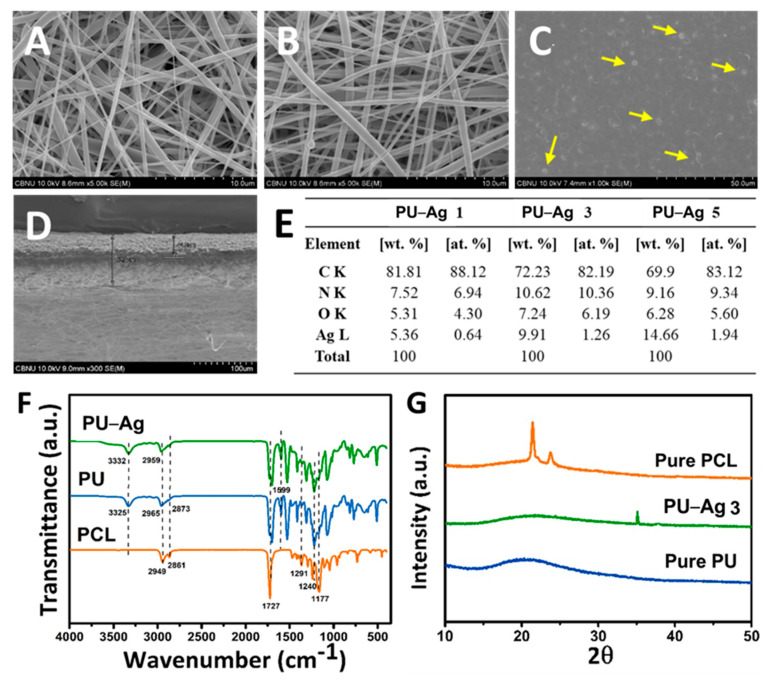
FE-SEM images of: (**A**) PCL nanofibers; (**B**) PU–PTX nanofibers; (**C**) PU–Ag film; and (**D**) cross-section of nanofibrous PU–PTX and PU-Ag film layer. (**E**) EDS analysis showing different concentration (1%, 3%, and 5%) of Ag in PU–Ag film. (**F**) FT-IR and (**G**) XRD results of PCL, PU, and PU–Ag 3.

Figure 2A–C shows the micrograph images of each layer: PCL and PU-PTX nanofibers and PU-Ag film. Uniformity in fiber thickness and smoothness as well as the beadless morphology of nanofibrous samples confirmed separate layers of PCL and PU were a suitable platform for controlled drug delivery. Figure 2D shows cross-sectional image of the drug-loaded nanofibers and the silver-containing PU film attached together. We confirmed that electrospinning the PU-based nanofibers on top of the PU-based film is a feasible technique to support adhesion between the two layers of the coating membrane. Furthermore, the EDS analysis of PU film loaded with AgNPs demonstrated the relative concentration of C, N, O, and Ag, thus proving a successful in situ formation of AgNPs on the surface of PU film as well as the EDS elemental mapping (Figure S1). Moreover, from the table we found that the wt.% of Ag increased from 5.36 to 14.66 as the concentration increase from 1% to 5%.

FT-IR spectra of pure PCL and PU and composite PU-Ag samples are represented in Figure 2F. PCL showed characteristic peaks at 1727 cm^−1^ (carbonyl stretching), 1240 cm^−1^ (asymmetric C-O-C stretching), 1190 cm^−1^ (O-C-O stretching), 1177 cm^−1^ (symmetric C-O-C stretching), 1291 cm^−1^ (C-O and C-C stretching in crystalline phase), 1162 cm^−1^ (C-O and C-C stretching in amorphous phase), 2949 cm^−1^ (asymmetric CH_2_ stretching), and 2861 cm^−1^ (symmetric CH_2_ stretching) [33].

To characterize the molecular nature of the pure and composite PU film mats, FT-IR spectra were taken and compared as shown in Figure 2F. The peaks of the PU film mat were assigned as follows: 3325 cm^−1^ (hydrogen bonded-NH stretching), 1727 cm^−1^ (carbonyl stretching), and 1599 cm^−1^ (C=C, benzene ring). The absorption bands related to asymmetric and symmetric CH_2_ stretching were observed at 2965 and 2873 cm^−1^, respectively, while various modes of CH_2_ vibrations were manifested in the range 1220–1413 cm^−1^. It is well-known that the absorption peaks concerning the amine and carbonyl groups are considered to evaluate the intermolecular hydrogen bonding affinity of the PU matrix. Similarly, the corresponding peaks of pure PU at 3325 cm^−1^ (-NH stretching) shifted to 3332 cm^−1^ in the composite PU film containing AgNPs, which indicates that the majority of NH groups in urethane linkages (HN-COO) participated in hydrogen bonding with the hydroxyl (OH) group capped on the surface of the metallic nanoparticles [30]. Moreover, a slight shift toward lower value of the peak at 2959 cm^−1^ are associated with asymmetric CH_2_ stretching band. Since AgNPs do not have absorption band in the infrared region, the FT-IR spectrum of both samples is almost identical except for the presence of the aforementioned bands shift. These findings confirm a strong chemical interaction between the inorganic nanoparticles and the organic polymer mat, which reveals the successful preparation of the composite mats.

The phase and purity of the materials are confirmed by the X-ray diffraction patterns. The XRD patterns of PCL nanofibers, PU film, and composite PU-Ag 3 film membranes are shown in Figure 2G. PCL sample exhibits two significant crystalline peaks at 2θ = 21.4° and 23.7° [29]. The diffraction pattern of the pure PU film displays a broad peak at 2θ = 21.3°, which is attributed to the amorphous phase of PU, while, in the case of PU-AgNPs film, two additional diffraction peaks were observed at 2θ = 35 and 37.8° along with the characteristic peaks of PU [30]. The reaction mechanism is proposed to be a simple and simultaneous process where silver nitrate is reduced to Ag(0) by aq. DMF due to formation of hydrogen ion [31]. The result obtained from XRD leads to the conclusion of successful reduction of AgNO_3_ and the formation of AgNPs on the surface of PU film.

### 3.2. In Vitro Drug Release Study

**Figure 3:**
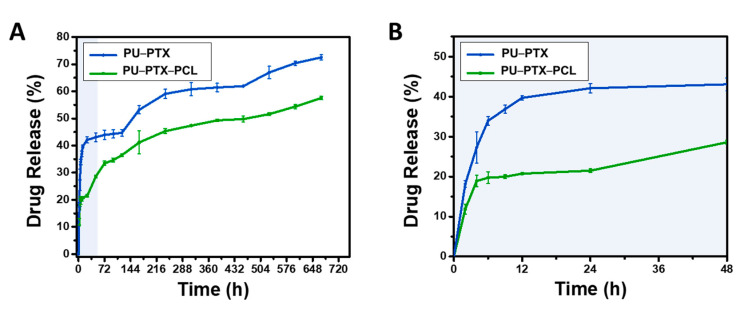
(**A**) The comparison of PTX release behavior between PTXloaded PU nanofibers enveloped with and without the PCL layer. (**B**) Initial 48 h measurement of PTX release profile. Three percent PTX is used in all samples.

Drug release behaviors of stent coating membrane PU-PTX-PCL was performed using UV spectrophotometer to measure the amount of PTX released in PBS after the fixed time. To evaluate whether the outer PCL layer can effectively function as a drug release barrier, we set PU-PTX as our control and compared the behavior. Therefore, in this analysis, the drug release curve of stent coating membrane was acquired at predetermined time (Figure 3). A significant burst release phenomenon is shown in the control sample after 24 h, releasing almost 40% of the drug compared to the 20% drug release in PU-PTX-PCL. The dramatic decrease in the initial burst in PU–PTX–PCL is an indication that the presence of PCL nanofibers enveloping around the drug-loaded layer is an effective method for slowing down the rapid drug release.

This result also indicates that it is much more difficult for water molecules to reach the drug particles when the drug-loaded layer is enveloped by the PCL nanofibers and PU film barriers on each side. We postulate that 20% of the initial burst in the PU-PTX-PCL reflects the dissolution of the drug aggregate particles on or near the perimeter of the PTX-loaded PU layer. After the initial burst of the drug aggregate particles on the surface of PU–PTX layer, the remaining drug entrapped in the membrane could only be accessed by the water molecules penetrating from the fibrous side since the other side is blocked by the PU film.

Consecutively, we observed that, after 28 days of the in vitro study, over 70% of PTX was released in the control while PU-PTX-PCL only released up to 57% of the drug content. Therefore, the results indicate that the outermost layer effectively serves the role of sustaining the release profile and inhibiting the initial burst release.

#### Mechanism of Drug Release

The in vitro release kinetics of PTX from PU was studied using zero- and first-order kinetic models (Table 1). The regression coefficient (R^2^) values obtained from the first-order kinetic model are greater than those from the zero-order kinetic model. The results reveal that the PTX release from PU nanofibers follows first order kinetics.

Korsemeyer–Peppas model is essential to explain the mechanism of drug release from a polymeric system. Our drug release data fit the Korsemeyer–Peppas model. This model describes whether the release profile follows Fickian diffusion, which can only be determined on the basis of the *n* value. The release exponent obtained from Korsemeyer–Peppas model is *n* = 0.243 (*n* > 0.5), implying that Fickian diffusion is the driving force for drug release.

### 3.3. Antibacterial Test

Many cases of biliary obstruction occur as a result of CCA, and using stents that pass through the bile duct is a palliative solution to this problem. However, the formation of biofilm in combination with a high density of biliary fluids is a common complication of stent insertion. Moreover, high incidence of bacterial infection occurs from biliary injury, bacterial adherence, and sludge, which cause biliary stent occlusion. Thus, we investigated the antibacterial activity of the AgNPs in the biliary stent coating.

**Figure 4:**
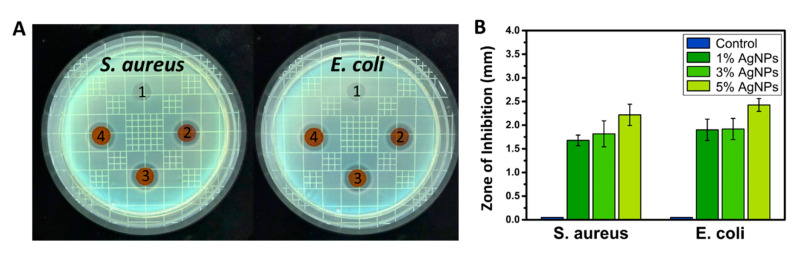
(**A**) Digital images and (**B**) zone of inhibition for antibacterial effect of PU films containing 0%, 1%, 3%, and 5% of AgNPs against *Staphylococcus aureus* and *Escherichia coli*.

After the biliary injury, multispecies consortia are detected in the stent system in most cases, with the combination of Gram-positive and Gram-negative bacteria giving a synergistic effect on the biofilm formation [32]. According to previous findings, *S. aureus* and *E. coli* are commonly found in the biofilm on biliary stents along with species of *Pseudomonas*, *Citrobacter*, and *Enterobacter* [16,33]. *S. aureus* is Gram-positive bacteria and can be identified using a commercially available *S. aureus* chromogenic medium. We found that the inhibition zone of *S. aureus* was significantly decreased in the AgNPs-immobilized group versus the control (Figure 4). From testing the antibacterial activity in the samples containing AgNPs, we also noticed a significant increase in the inhibition zone of *E. coli*. One, three and five percent of AgNPs cultured with *E. coli* showed a gradual increase in the inhibition zone compared to pure PU film. Notably, after 24 h of culturing the PU-Ag samples, the survival ratio of *E. coli* continuously decreased compared to that of the control, suggesting that AgNPs exhibited a highly efficient antibacterial activity for both short- and long-term periods. The data suggest that the PU-Ag film possessed a strong broad-spectrum antibacterial function, and this can be explained by the ability of the metallic AgNPs to change the metabolite path of the bacterial cells so that the Ag ions can undergo an ion exchange between the thiol and imidazole groups in the bacteria, resulting in a DNA malfunction that inhibits the proliferation ability of the bacteria [34,35].

### 3.4. In Vitro Cytotoxicity Test

**Figure 5:**
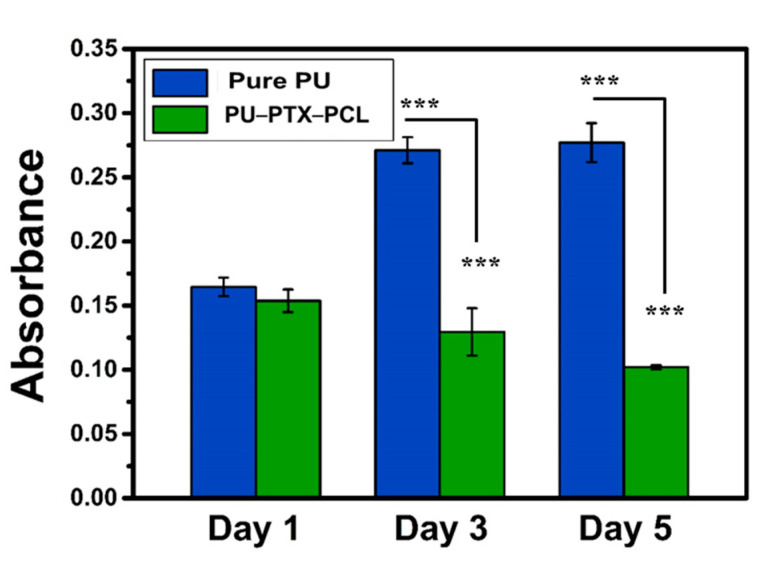
CCK-8 assay of CT26 cultured on pure PU and PU-PTX-PCL. The test was performed on Days 1, 3, and 5, and the drug content in PU-PTX-PCL samples were maintained to 3%. *p*-values were calculated using *t*-tests (*** *p* < 0.001).

To explore the application of PU-PTX-PCL in chemotherapy, in vitro cell culture test was used to study their potential toxicity on cancer cells using CCK-8 kit. Cell viability assays are vital to the preliminary evaluation of biomaterials and biomedical implants for potential bio-applications, in order to confirm the cytotoxicity of the fabricated materials. Thus, CCK assay was performed with CT26 colon cancer cells for different samples of PU and PU-PTX-PCL at different interval time of one, three, and five days, as shown in Figure 5. After the first day of incubation, cellular responses in PU-PTX-PCL showed only a slight decrease in cell viability. On Days 3 and 5 of incubation, however, PU-PTX-PCL showed a significantly higher cytotoxicity compared to pure PU, suggesting the potential efficacy of PTX in gastrointestinal tumor growth inhibition.

## 4. Conclusions

In this study, we presented a novel tri-layer membrane that consists of multi-functions for prolonging the stent patency. The inner layer of AgNPs-immobilized PU film has bacterial biofilm-suppressing effect. The middle layer is composed of electrospun PU nanofibers loaded with PTX. Lastly, the outer layer of PCL nanofibers was electrospun on top of the drug-loaded PU membrane to control the initial burst release of PTX. The tri-layer membrane shows a sustained release of PTX over 28 days, and we confirmed that the release profile followed Fickian diffusion mechanism. Our results reveal that AgNPs-loaded samples have a significant antibacterial effect against *E. coli* and *S. aureus* due to the silver particles. The in vitro cell culture test showed high cytotoxic effect of PTX towards colon cancer cells as defined by the gradual decrease in cell viability compared to the pure PU mats. Based on the results, our fabricated material shows long-term patency and curative potential. We anticipate that the contributions made here should offer practical advantages and improved clinical outcomes.

## Figures and Tables

**Table 1:** In vitro drug release kinetics of PTX from PU-PTX and PU-PTX-PCL.

Kinetics Models	Parameter	PU–PTX	PU–PTX–PCL
Zero order	R^2^	0.821	0.838
First order	R^2^	0.914	0.896
Korsemeyer–Peppas	R^2^	0.902 (*n* = 0.243)	0.975 (*n* = 0.18)

## Supplementary Materials

The following are available online at https://www.mdpi.com/2079-4991/11/2/486/s1, Figure S1: EDS elemental mapping showing the composition PU films containing 1, 3, and 5% of AgNPs. **A** is corresponding to (C), B is corresponding to (N), C is corresponding to (O), D is corresponding to (Ag) and (E) is corresponding to merged image from A to D., Figure S2. The comparison of PTX release behavior between PTX-loaded PU nanofibers enveloped with and without the PCL layer. The release profile confirms that the PCL layer effectively controls the initial burst release.
