# Supplementary material for: A Novel Design of Tri-Layer Membrane with Controlled Delivery of Paclitaxel and Anti-Biofilm Effect for Biliary Stent Applications
Source: Nanomaterials (Basel). 2021 Feb 14;11(2):486. doi: 10.3390/nano11020486 (PMC7918081; doi:10.3390/nano11020486)
Supplement: Supplementary file 1 [file nanomaterials-11-00486-s001.pdf]

A Novel Design of Tri-Layer Membrane with Controlled Delivery of Paclitaxel and Anti-Biofilm Effect for Biliary Stent Applications

Abdelrahman I. Rezk [1,2,†], Jeesoo Park [1,2,†], Joon Yeon Moon [1,2], Sunny Lee [1,2], Chan Hee Park [2,3,4,*] and Cheol Sang Kim [2,3,4,*]

[1] Department of Bionanosystem Engineering, Graduate School, Jeonbuk National University, Jeonju 561-756, Republic of Korea

[2] Department of Bionanotechnology and Bioconvergence Engineering, Graduate School, Jeonbuk National University, Jeonju 561-756, Republic of Korea

[3] Division of Mechanical Design Engineering, Jeonbuk National University, Jeonju 561-756, Republic of Korea

[4] Eco-Friendly Machine PartsDesign Research Center, Jeonbuk National University, Jeonju, Republic of Korea

*Corresponding authors:

Tel: +82 63 270 4284, Fax: +82 63 270 2460

Cheol Sang Kim (chskim@jbnu.ac.kr)

Chan Hee Park (biochan@jbnu.ac.kr)

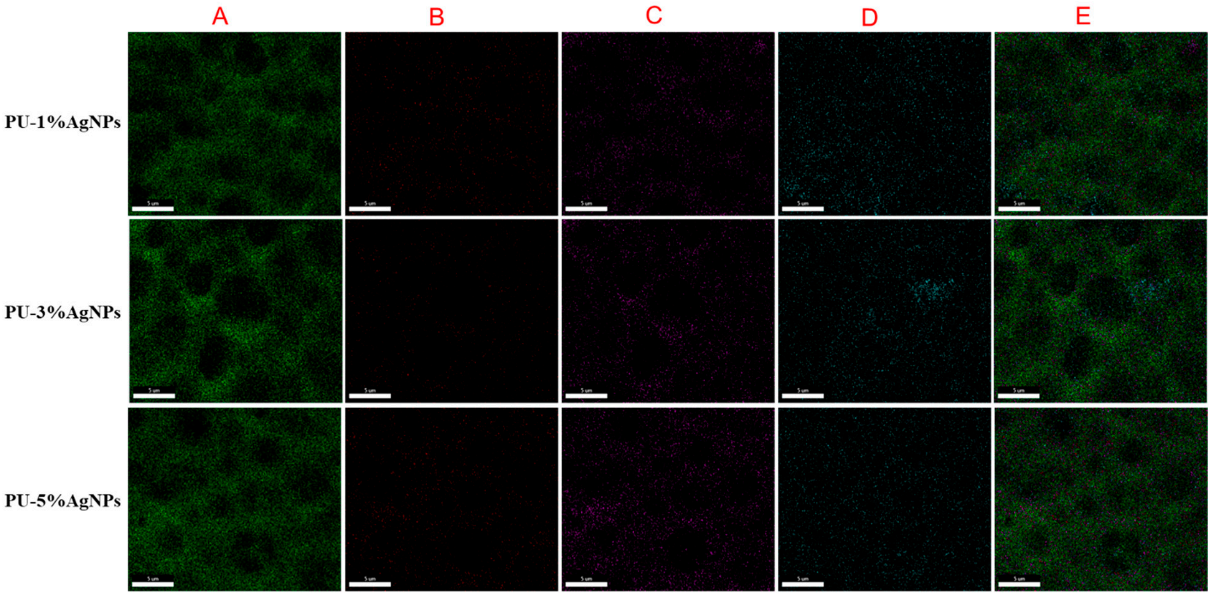

Figure S1: EDS elemental mapping showing the composition PU films containing 1, 3, and 5% of AgNPs. A is corresponding to (C), B is corresponding to (N), C is corresponding to (O), D is corresponding to (Ag) and (E) is corresponding to merged image from A to D.

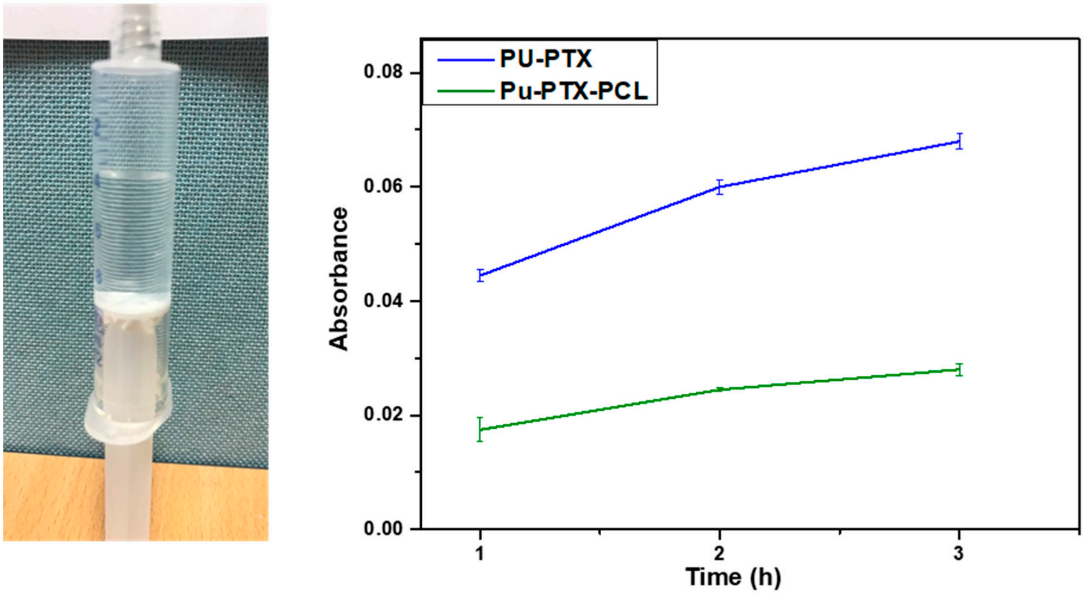

Figure S2. The comparison of PTX release behavior between PTX-loaded PU nanofibers enveloped with and without the PCL layer. The release profile confirms that the PCL layer effectively controls the initial burst release.
